# MLVA Typing of *Streptococcus pneumoniae* Isolates with Emphasis on Serotypes 14, 9N and 9V: Comparison of Previously Described Panels and Proposal of a Novel 7 VNTR Loci-Based Simplified Scheme

**DOI:** 10.1371/journal.pone.0158651

**Published:** 2016-07-08

**Authors:** Natália S. Costa, Tatiana C. A. Pinto, Vânia L. C. Merquior, Luciana F. S. Castro, Filomena S. P. da Rocha, Jaqueline M. Morais, José M. Peralta, Lúcia M. Teixeira

**Affiliations:** 1 Instituto de Microbiologia Paulo de Góes, Universidade Federal do Rio de Janeiro, Rio de Janeiro, RJ, Brasil; 2 Faculdade de Ciências Médicas, Universidade do Estado do Rio de Janeiro, Rio de Janeiro, RJ, Brasil; University of Mississippi Medical Center, UNITED STATES

## Abstract

*Streptococcus pneumoniae* remains as an important cause of community-acquired bacterial infections, and the nasopharynx of asymptomatic carriers is the major reservoir of this microorganism. Pneumococcal strains of serotype 14 and serogroup 9 are among the most frequently isolated from both asymptomatic carriers and patients with invasive disease living in Brazil. Internationally disseminated clones belonging to such serotypes have been associated with the emergence and spread of antimicrobial resistance in our setting, highlighting the need for epidemiological tracking of these isolates. In this scenario, Multiple Loci VNTR Analysis (MLVA) has emerged as an alternative tool for the molecular characterization of pneumococci, in addition to more traditional techniques such as Multi-Locus Sequence Typing (MLST) and Pulsed-Field Gel Electrophoresis (PFGE). In the present study, 18 VNTR loci, as well as other previously described reduced MLVA panels (7 VNTR loci), were evaluated as tools to characterize pneumococcal strains of serotypes 14, 9N and 9V belonging to international and regional clones isolated in Brazil. The 18 VNTR loci panel was highly congruent with MLST and PFGE, being also useful for indicating the genetic relationship with international clones and for discriminating among strains with indistinguishable STs and PFGE profiles. Analysis of the results also allowed deducing a novel shorter 7 VNTR loci panel, keeping a high discriminatory power for isolates of the serotypes investigated and a high congruence level with MLST and PFGE. The newly proposed simplified panel was then evaluated for typing pneumococcal strains of other commonly isolated serotypes. The results indicate that MLVA is a faster and easier to perform, reliable approach for the molecular characterization of *S*. *pneumoniae* isolates, with potential for cost-effective application, especially in resource-limited countries.

## Introduction

Members of the species *Streptococcus pneumoniae*, usually referred as pneumococci, colonize the human nasopharynx and are frequently associated with invasive and non-invasive infections, particularly among children, the elderly and immunocompromised hosts [1].

Antimicrobial resistant pneumococci have been emerging worldwide since the 1990’s [2]. Although more than 90 different capsular types have already been described among pneumococcal strains [3], antimicrobial resistance is usually restricted to a smaller number of serogroups or serotypes, among which serotype 14 and serogroup 9 are prominent due to their frequent association with both invasive pneumococcal disease (IPD) and asymptomatic nasopharyngeal carriage [4–9].

In Brazil, serotypes 14, 9N and 9V have been described among the five types most commonly isolated from IPD in the pre-vaccination era [10–15] and also a few years after implementation of the first pneumococcal conjugate vaccine (10-valent, PCV10) in 2010 [16, 17]. Moreover, emergence of antimicrobial resistant pneumococci, especially penicillin non-susceptible pneumococci (PNSP), in our country has already been linked to the dispersion of international (Spain^9V^-3 ST156, Tennessee^14^-18 ST67 and England^14^-9 ST9) and regional clones (ST5401) belonging to such serotypes [18].

Such clones are usually characterized by using PFGE (Pulsed-Field Gel Electrophoresis) and MLST (Multilocus Sequence Typing). Although these methods represent the “gold standard” for molecular epidemiology purposes, they can be expensive, time-consuming or, in the case of PFGE, generate results which are difficult to compare among different laboratories. In this context, MLVA (Multiple loci VTNR [Variable-Number of Tandem Repeat] Analysis) emerges as an alternative method that would combine the promptness of MLST with the high discriminatory power of PFGE.

In a first investigation on the use of MLVA for typing pneumococci a set of 18 VNTR loci was identified [19]. Subsequent studies have proposed to use subsets of this collection, comprising 7–10 VNTR loci to be included in more practical approaches [20–23]. Most of these previous studies have applied MLVA for typing pneumococcal isolates recovered from IPD outbreaks [19, 20] or from IPD cases registered during short periods of time [22–24]. In addition, only 3% [23] to 25% [22] of the isolates included in previous studies belonged to serotypes 14, 9N and 9V.

In the present study, the genetic diversity among *S*. *pneumoniae* belonging to serotypes 14, 9N and 9V isolated in Brazil was evaluated by MLVA, in comparison with PFGE and MLST, using all the 18 VNTR loci originally identified. An *in silico* comparison was also performed with reduced panels previously described. From the resulting data, a new panel of seven loci presenting high discriminatory power was evaluated and proposed for typing pneumococci of different serotypes.

## Materials and Methods

### Bacterial strains

A total of 174 *S*. *pneumoniae* strains have been investigated in the present study. They were divided in 2 sets. One set comprised 87 isolates evaluated in the first part of the study, which included sixty isolates belonging to serotype 14, nine to serotype 9N and eighteen to serotype 9V. The isolates were selected in order to represent the genetic diversity previously observed by MLST [18], and belonged to 25 different STs. They were mainly recovered from patients with IPD (66 strains), or from patients with non-invasive diseases (6 strains), as well as asymptomatic carriers (15 strains), living in Brazil, between 1991 and 2009

The other set was also composed by 87 pneumococcal isolates, which were included in order to challenge a proposed alternative 7 VNTR loci panel, based on the results of the first part of the study. Isolates composing this second set were randomly selected from our culture collection and belonged to serotypes other than 14, 9N and 9V. The characteristics of all the isolates included in the study are presented in S1 Table.

Each strain had previously been identified and characterized on the basis of Gram-staining, colony morphology, hemolytic activity, optochin susceptibility and bile solubility [25]. The capsular types were determined either by multiplex PCR [26] or by standard Quellung reaction [27].

In addition, eight PMEN clones represented by reference strains were included in the present study (Spain^9V^-3 ST156, Spain^14^-5 ST 18, England^14^-9 ST9, CSR^14^-10 ST20, Tennessee^14^-18 ST67, Denmark^14^-32 ST230, Netherlands^14^-35 ST124 and Netherlands^15B^-37 ST199).

### PFGE analysis

PFGE analysis was performed with the first set of strains (87 isolates belonging to serotypes 14, 9N and 9V) and the eight PMEN clones.

Genomic DNA was prepared in agarose plugs as described by Pinto et al. [28]. After macrorestriction using *Sma*I (New England Biolabs, Ipswich, MA, USA), the fragments were separated in a CHEF-DR III system (Bio-Rad, Hercules, CA, USA) by using the parameters recommended by the PMEN [http://www.pneumogen.net/pmen/index.html]. The restriction profiles were named according to an in-house nomenclature; when they were associated with a specific antimicrobial resistance trait of importance, their denomination included "Pen" for penicillin or "Ery" for erythromycin resistance. BioNumerics software version 7.5 (Applied Maths, Ghent, East Flanders, Belgium) was used to create dendrograms by applying the unweighted-pair group method with arithmetic mean (UPGMA) and the Dice similarity coefficient, with optimization and position tolerance settings of 0.5% and 1.3%, respectively. Profiles showing more than 70% of similarity were clustered in the same clonal complex (CC).

### MLVA

MLVA was performed with all 174 clinical isolates and the eight PMEN clones.

Bacterial DNAs were obtained by using the Chelex^®^ 100 resin (Bio-Rad, Hercules, CA, USA) as described earlier [28], and eighteen VNTRs (Spneu15, Spneu17, Spneu19, Spneu25, Spneu26, Spneu27, Spneu31, Spneu32, Spneu33, Spneu34, Spneu35, Spneu36, Spneu37, Spneu38, Spneu39, Spneu40, Spneu41, Spneu42) were amplified by uniplex PCR reactions as previously described [19]. Each reaction contained 10 ng of DNA, 1 U of *Platinum Taq* DNA polymerase, 1 X PCR buffer, 200 micromolar of each dNTP, 1.5 mM of MgCl_2_, and 0.3 micromolar of each primer in final volume of 15 microliters. All reagents were purchased from Invitrogen (Carlsbad, CA, USA). Amplifications were performed in Veriti 96-Well Thermal Cycler (Applied Biosystems, Life Technologies, Carlsbad, CA, USA) with 30 cycles of denaturation at 94°C for 30s, primer annealing at 60°C for 30s, and elongation at 72°C for 45s. The number of repeats in each locus was determined by estimating the size of amplification products separated in 2% (for most of the VNTR loci) or 4% (for loci Spneu41 and Spneu42) agarose (UltraPure Agarose, Invitrogen, Carlsbad, CA, USA) gels, in which were included, respectively, 100 bp or 10 bp DNA ladders (Invitrogen). *S*. *pneumoniae* ATCC BAA-255 (R6) strain was included in each PCR reaction for quality control.

MLVA types (MT) and their respective CCs were numbered according to an in-house nomenclature. In addition, the *S*. *pneumoniae* MLVA database hosted at www.mlva.eu [last accessed on March 12^th^ 2016] and comprising 17 out of the 18 loci previously described by Koeck et al. [19] was searched for the occurrence of identical or similar allelic profiles (up to five different alleles among the 17 VNTR loci) according to the respective instructions.

Also, a public database comprising metadata and MLVA allelic profiles of all strains analyzed in the present study was created and deposited at http://mlva.u-psud.fr/ under the name “Pneumo MLVA Brazil”.

Analysis of MLVA numerical profiles and construction of dendrograms and minimum spanning trees (MST) were performed by using BioNumerics v7.5 (Applied Maths). MTs sharing at least 70% of similarity (12 or more identical loci among the total of 18, and 5 or more identical loci among the total of 7) were included in the same CC.

Amplification products of randomly selected alleles of each VNTR locus were purified using ExoSAP-IT^®^ (USB Affymetrix, Cleveland, OH, USA) and sequenced using an ABI 3130 Genetic Analyzer (Applied Biosystems). Sequences were edited and aligned with the BioEdit software version 7.0.9.0 [29], and deposited in the GenBank database under accession numbers KP275552-KP275574. BLAST searching [http://blast.ncbi.nlm.nih.gov/Blast.cgi] was performed to detect similar sequence records.

### Statistical analysis

The discriminatory powers of MLVA, MLST and PFGE techniques and of each one of the 18 VNTR loci evaluated were predicted using the Simpson’s index of diversity (SID), by calculating the 95% confidence intervals (95% CI) [30, 31]. Congruence among MLVA, MLST and PFGE methods, and among the different panels of loci evaluated, was assessed by using the Adjusted Wallace coefficient (AW) [32]. SID and AW were calculated with the online tool Comparing Partitions [www.comparingpartitions.info].

### Ethics statement

No human participants were involved directly in this study and no clinical information was used. Therefore, human ethics clearance and informed consent were not specifically required. The bacterial isolates included in the present work were sent to our laboratories for typing characterization or were obtained during previous surveillance studies conducted in compliance with Good Clinical Practice and the Declaration of Helsinki of the World Medical Association and approved by the ethics committees of the different institutions involved.

## Results and Discussion

MLST had previously classified the 87 serotypes 14, 9N and 9V pneumococcal isolates included in the present study in 25 STs, distributed in four CCs and six singletons [18]. Those four clusters were represented by CC156, associated with the international clone Spain^9V^-3 ST156; CC66, associated with Tennessee^14^-18 ST67; CC15, associated with England^14^-9 ST9; and CC5401, which represented a novel and probably regional clone. Such clones have been circulating and were associated with the emergence of antimicrobial resistance among pneumococci recovered in our setting over a period of 23 years [18].

**Fig 1:**
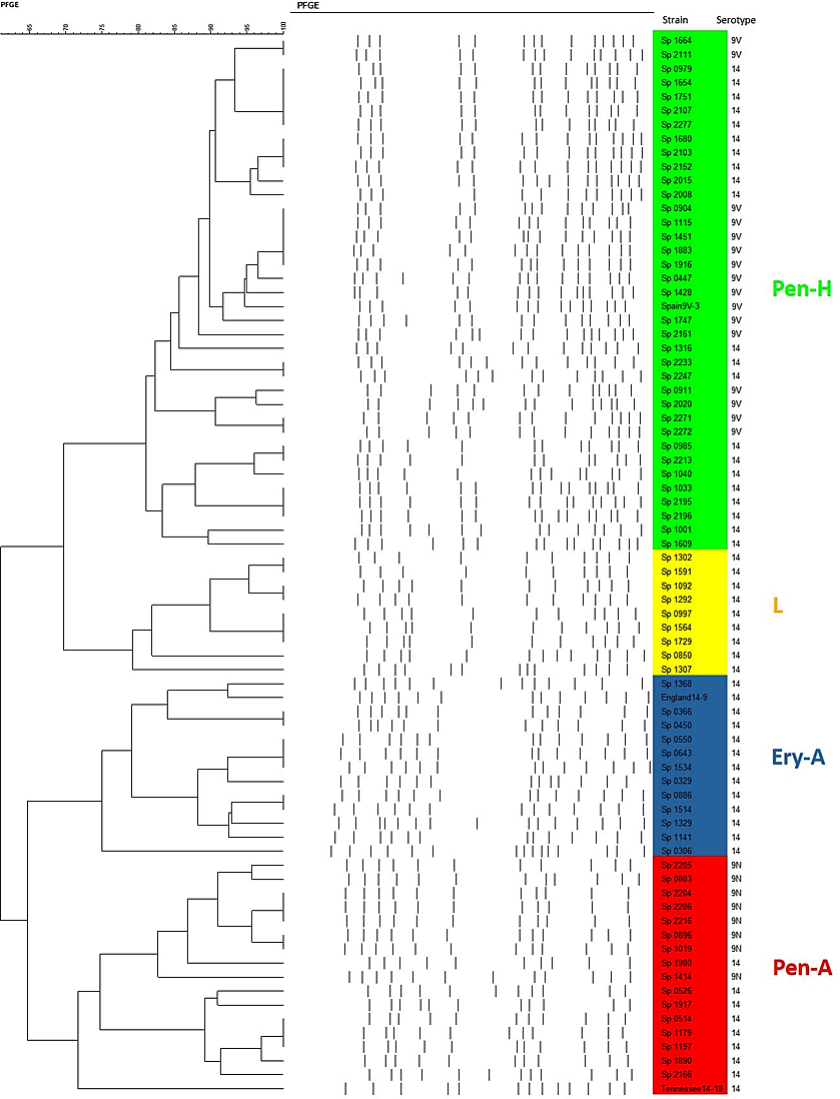
Genetic relationship observed by PFGE among *Streptococcus pneumoniae* isolates belonging to the four major clusters detected in the present study (namely Pen-H, L, Ery-A and Pen-A), including related PMEN clones. Clonal complexes are distinguished by colors.

PFGE analysis revealed 44 different profiles distributed in four CCs and seven singletons among the 87 pneumococcal isolates (Fig 1). A single cluster, named Pen-H, included 43.7% (38/87) of the strains, and was associated with Spain^9V^-3 ST156. The second major cluster, named Pen-A, comprised 19.5% (17/87) of the isolates and was associated with Tennessee^14^-18 ST67. The third cluster, Ery-A, included 14.9% (13/87) of the isolates and was associated with England^14^-9 ST9. The occurrence of this PFGE CC among Brazilian pneumococcal isolates has already been documented by Mendonça-Souza et al. [33]. The fourth, cluster L, included 11.5% (10/87) of the isolates, and comprised isolates belonging to the new MLST clone CC5401.

**Fig 2:**
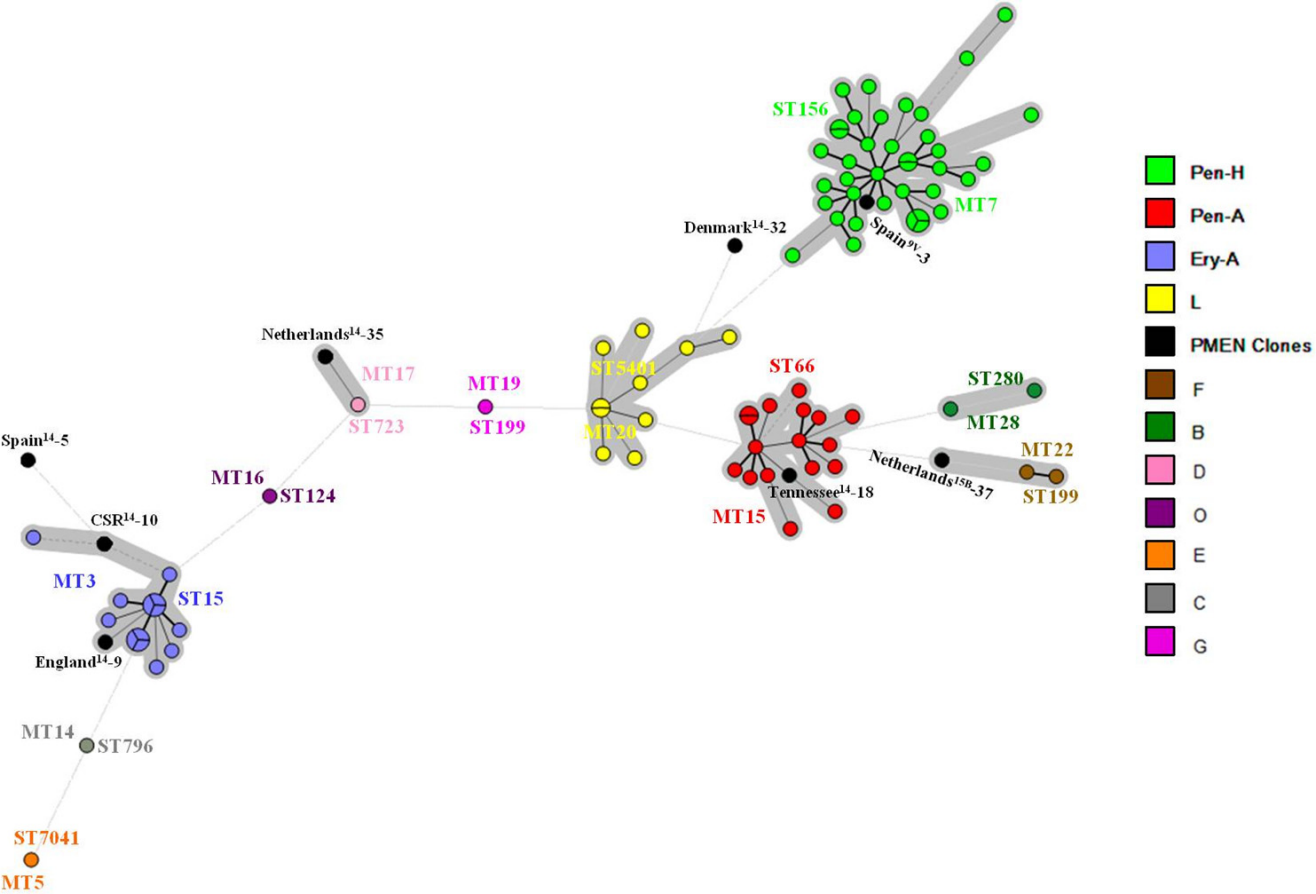
Minimum Spanning Tree showing the genetic relationship by MLVA among 87 *Streptococcus pneumoniae* strains of serotypes 14, 9N and 9V and the eight PMEN clones included in the present study using the 18 VNTR loci described by Koeck et al. [19]. Legend is according to PFGE clonal complex. Different colors represent different MLVA types, and the size of the circle indicates the number of isolates belonging to the same type. MLST and MLVA complexes and PMEN clones are described. Clonal complexes are highlighted with a grey shadow. Lines linking the circles denote the genetic distance as follows: heavy short lines connect single locus variants (SLVs), thin longer lines connect double-locus variants (DLVs) and dotted lines indicate difference by 3 or more loci.

MLVA using all the 18 VNTR loci investigated in the present study revealed the occurrence of 77 MTs among the 87 pneumococcal isolates belonging to serotypes 14, 9N and 9V, which were distributed in six CCs and five singletons (Fig 2). Four of these CCs were distinguishable due to their prevalence and congruence with MLST and PFGE results. The predominant, named cluster 7, comprised 43.7% (38/87) of the isolates, and was associated with Spain^9V^-3 ST156 complex and PFGE cluster Pen-H. The second most prevalent, cluster 15, encompassed 19.5% (17/87) of the isolates, and was associated with Tennessee^14^-18 ST67 complex and PFGE cluster Pen-A. Cluster 3 included 14.9% (13/87) of the isolates, and was associated with England^14^-9 ST9 complex and PFGE cluster Ery-A. MLVA cluster 20 included 11.5% (10/87) of the isolates, and was associated with MLST CC5401 and PFGE cluster L.

A high level of congruence among MLST, PFGE and MLVA results was detected within each one of these four major CCs, suggesting the potential application of MLVA for a faster identification of pneumococcal clones. Still, higher diversity indexes were observed for MLVA when compared to PFGE and MLST (Table 1). Noteworthy, the SID (99.3%) observed within the 38 CC156 isolates included in the present study was much higher than the diversity index (90.39%) previously detected among 56 strains belonging to the same CC recovered in Poland between 2003 and 2005 [7].

**Table 1:** Indexes of diversity observed within the four major clonal complexes (CCs) detected among 87 *Streptococcus pneumoniae* isolates belonging to serotypes 14, 9N and 9V by comparing three different typing methods (MLVA, PFGE and MLST).

CC (number of strains)	Typing Method^a^	Number of types^b^	SID^c^ (95% CI)
**MLVA 7 PFGE Pen-H MLST CC156 (38 strains)**	MLVA^18^	34	0.993 (0.983–1.000)
	PFGE	9	0.802 (0.708–0.897)
	MLST	6	0.555 (0.404–0.706)
**MLVA 15 PFGE Pen-A MLST CC66 (17 strains)**	MLVA^18^	16	0.993 (0.974–1.000)
	PFGE	5	0.779 (0.654–0.905)
	MLST	6	0.647 (0.416–0.879)
**MLVA 3 PFGE Eri-A MLST CC15 (13 strains)**	MLVA^18^	9	0.923 (0.838–1.000)
	PFGE	7	0.795 (0.592–0.997)
	MLST	2	0.154 (1.000–0.404)
**MLVA 20 PFGE L MLST CC5401 (10 strains)**	MLVA^18^	9	0.978 (0.927–1.000)
	PFGE	5	0.844 (0.743–0.946)
	MLST	5	0.667 (0.357–0.977)
**All**^**d**^ **(87 strains)**	MLVA^18^	77	0.997 (0.993–1.000)
	PFGE	33	0.948 (0.925–0.971)
	MLST	25	0.881 (0.838–0.924)

^a^MLVA^18^, MLVA scheme based on 18 VNTR loci

^b^Number of profiles observed for each typing method used

^c^SID, Simpson’s Index of Diversity; 95% CI, Confidence Interval

^d^Represented by the first set of isolates of the present study (87 strains belonging to serotypes 14, 9N or 9V)

MLVA CC7 (MLST CC156 and PFGE Pen-H) was associated with high-level penicillin non-susceptibility. In the MLVA database searched, which currently contains 1818 pneumococcal strains and 1087 different genotypes (Last update on February 25^th^ 2011), one record (namely genotype 708) was identical to the most prevalent MT among CC7 strains of the present study. Genotype 708 was represented by a penicillin-resistant isolate belonging to serotype 9V and ST156, recovered from a nasopharyngeal carrier in Poland in 2005 [7]. Likewise, MT7 identified in the present study comprised PNSP isolates belonging to serotype 9V and ST156, recovered from invasive infections between 2001 and 2004, in Brazil, showing that the same genotype was circulating in distant geographical areas during a short period of time. In addition, several other records in the database presented genetic similarity (up to 5 different loci) with MLVA CC7 profiles, and they also belonged to serotypes 14 or 9V, ST156, and were non-susceptible to penicillin, being mainly recovered in France and Poland during 2003 and 2011. These findings indicate that MLVA can successfully identify and cluster together isolates associated with Spain^9V^-3 ST156 complex regardless of geographical origin and period of time.

Previously, we have found that the introduction of ST156 in our setting occurred in the mid 1990’s, and was the major force behind the emergence of penicillin resistance in Brazil [18]. We have also observed that this event was preceded by the emergence of trimethoprim/sulfamethoxazole resistance and by the dissemination of ST162, which is the probable penicillin-susceptible ancestor of ST156 and is generally included in the same CC by MLST [18]. In the present study, MLVA was useful for distinguishing between penicillin-non-susceptible ST156 isolates and penicillin-susceptible ST162 isolates. Particularly, in locus Spneu40, 2–3 repeats were found in ST162 representatives while a number of 8 repeats was observed among the ST156 isolates investigated in the present study. Likewise, van Cuyck et al. [22] observed that MLVA could successfully discriminate between these two STs among invasive isolates recovered in UK; however, Spneu40 was not included in the panel of loci evaluated. Once it is apparently possible to associate the number of repeats in certain VNTR loci with certain advantageous characteristics and since neutral genetic markers, such as VNTR loci, usually evolve faster [7], the detection of variations in such loci could be useful to predict the emergence of virulence and resistance traits among pneumococcal clones.

MLVA CC15 (MLST CC66 and PFGE Pen-A), in turn, was associated with low-level penicillin non-susceptibility. In the MLVA database searched, no records were found to be identical to any MT comprised by this CC; however, genotypes 725, 751 and 803 presented up to 3 different loci with certain CC15 profiles detected in the present study. Such genotypes were represented by isolates belonging to serotype 9N recovered in France during 2007 and 2009, showing that this CC is also widespread. However, no data on ST of such genotypes were available in the database, being hard to predict if MLVA would also successfully identify and cluster together isolates related to Tennessee^14^-18 ST67 complex recovered in different parts of the world.

MLVA CC3 (MLST CC15 and PFGE Ery-A) was associated with erythromycin resistance. Likewise, in the MLVA database, the closest match (4 different loci) was genotype 817, represented by an erythromycin-resistant isolate belonging to serotype 14, recovered in France in 2009. No data on ST of such genotype, however, was available in the database. This cluster is known to be associated with the dispersion of erythromycin resistance among pneumococci in Brazil since the 1990’s [18, 33]. In addition to England^14^-9 ST9, MLVA and PFGE also revealed that this cluster shows genetic relationship with the PMEN clone CSR^14^-10 ST20 (Fig 2).

Unlike other STs, isolates belonging to the PMEN clone Netherlands^15B^-37 ST199 included in the present study were classified in two distantly related MTs (19 and 22) (Fig 2), sharing only 9 identical alleles out of the 18 loci analyzed, which was corroborated by PFGE results. Each MT could be associated with different characteristics; while MT19 was associated with IPD, PNSP and tetracycline resistance, MT22 was associated with carriage, penicillin susceptibility and resistance to trimethoprim/sulfamethoxazole. This last MT was also closer to the profile observed for the related PMEN clone itself (Fig 2). These results indicate that, although derived from a common ancestor, ST199 strains circulating in Brazil might represent at least two independently evolved genotypes showing different adaptive characteristics. Such observation was only possible by using an approach more discriminatory than MLST, such as MLVA. Interestingly, such ST199 isolates of the present study represent serotype 14 variants of this clone, which is known to be more associated with serogroups 15 and 19 [http://pubmlst.org/spneumoniae/ Last accessed on March 12^th^ 2016]. Such unusual variants have been previously speculated to have been generated by capsular recombination with CC156 strains [18]. Indeed, PFGE and MLVA revealed that such strains were genetically more closely related to serotype 15B strains than to the other serotype 14 strains analyzed (data not shown), suggesting that MLVA also has potential to identify capsular switching events, as previously observed by others [22, 23]. Moreover, in the MLVA database searched, the closest matches to MTs 19 and 22 (4–5 different loci) were genotypes represented by serotype 19A isolates belonging to ST199.

On the other hand, no similar records were found in the MLVA database consulted for MLVA CC20 profiles and other sporadic STs included in the present study, suggesting that these clones have a limited circulation and supporting the previous observation of a regional origin for CC5401 [18].

Sequences of MLVA alleles of all 18 loci were consistent with what was expected for each VNTR locus, with percent matches to the reference strain R6 ranging from 83% to 100%, suggesting that PCR amplification had occurred properly. Additionally, as previously observed [19], sequences representing the locus Spneu26 showed a peculiar configuration, with an unusual 48-bp repeat presenting low internal homogeneity inserted inside the usual 51-bp repeats. Sequencing also confirmed the presence of an intermediate size allele containing 2.5 repeats in locus Spneu26.

Null MLVA alleles (coded “0.0” in this study), meaning that no amplification product was observed even after several PCR amplification attempts, were observed for 3 strains in Spneu19, 33 strains in Spneu25, 1 strain in Spneu34, 9 strains in Spneu36, and 3 strains in Spneu42. Loci Spneu19, Spneu25 and Spneu36 were already reported as being absent in some pneumococcal isolates [7, 19, 23], highlighting the lack of Spneu36 among Polish serotype 14/9V strains [7]. Since Spneu19 is inserted in the *pcp*A gene [19], which encodes a choline-binding protein [34], the absence of such VNTR locus might be linked with the absence of the *pcp*A gene itself. Spneu36, in turn, is fused in gene trz*A* in the strain R6 encoding the N-ethylammeline chlorohydrolase, a Atz/Trz family protein [19], but not enough is known to link the absence of such VNTR locus with the absence of *trz*A gene. On other hand, null alleles in Spneu25 have been previously associated with the presence of insertion sequence (IS) elements in this locus, which can generate long amplicons (>2000bp) [19, 23] that would not be detected properly depending on conditions used for PCR and agarose gel electrophoresis.

On the other hand, MLVA alleles coded "0.1" were attributed to loci without a VNTR unit but with an observed PCR product reflecting amplification of the flanking regions. Only locus Spneu38 generated such kind of amplicon, represented by 19.5% (17/87) of the strains, all of them belonging to MLVA CC15, including the international clone Tennessee^14^-18 ST67. Previously, Koeck et al. [19] showed that around 3% of the strains investigated, which belonged to different serotypes, had alleles “0.1” for this same locus.

The hypothetical absence of VNTR loci in the bacterial genome is not surprising since BOX elements are supposed to be highly variable and not essential for bacterial survival, and the specific functions of these elements are still unknown. However it was shown that VNTRs and BOX elements can play a role in bacterial competence and virulence and can impact the expression of genes downstream [35, 36]. Nevertheless, the absence of certain VNTR loci among a number of pneumococcal strains does not necessarily exclude these markers as putative options to be included in a universal panel of loci for this species, since this feature seems to be associated with specific serotypes and does not influence the discriminatory power.

Regarding the eight PMEN clones included in this study, eight different genotypes were obtained by MLVA, as expected (Fig 2). In the MLVA database searched, only the allelic profiles of Spain^14^-5 ST18 and CSR^14^-10 ST20 did not show any similar matches. However, except for Spain^9V^-3 ST156 matches, no data on ST of matching genotypes with the other PMEN clones were available, being hard to predict if MLVA profiles detected among PMEN clones in the present study would be reproducible in other parts of the world.

In general, MLVA had a high discriminatory power, displaying a diversity index higher than PFGE and MLST (Table 1), indicating its usefulness as a tool for molecular epidemiological studies. Also, a high level of congruence between MLVA, MLST and PFGE was observed (Table 2), showing the good correlation of MLVA with “gold standard” techniques. Likewise, Elberse and colleagues [21] observed high congruence between MLVA and MLST results among pneumococcal strains belonging to a variety of serotypes recovered from asymptomatic carriers in Portugal, generating a probability of 87.4% for two isolates belonging to the same MT sharing the same ST. Noteworthy, the index observed in the present study using all the 18 VNTR loci was even higher, with a probability of 91.3% for two isolates belonging to the same MT sharing the same ST (Table 2).

**Table 2:** Congruence levels (Adjusted Wallace Coefficient) among three typing methods evaluated in the present study (MLVA, PFGE and MLST), regarding both specific profiles and clonal complexes (CCs)^a^, for 87 *Streptococcus pneumoniae* isolates belonging to serotypes 14, 9N and 9V.

	MT^b^	MLVA CC	PFGE profile	PFGE CC	ST^c^	MLST CC
**MT**		1.000 (1.000–1.000)	0.432 (0.178–0.686)	1.000 (1.000–1.000)	0.913 (0.825–1.000)	1.000 (1.000–1.000)
**MLVA CC**	0.010 (0.000–0.028)		0.158 (0.077–0.239)	1.000 (1.000–1.000)	0.388 (0.252–0.523)	1.000 (1.000–1.000)
**PFGE profile**	0.028 (0.000–0.082)	1.000 (1.000–1.000)		1.000 (1.000–1.000)	0.456 (0.320–0.592)	1.000 (1.000–1.000)
**PFGE CC**	0.010 (0.000–0.028)	1.000 (1.000–1.000)	0.158 (0.077–0.239)		0.388 (0.252–0.523)	0.543 (0.404–0.682)
**ST**	0.024 (0.000–0.058)	0.994 (0.988–1.000)	0.184 (0.090–0.279)	0.994 (0.998–1.000)		1.000 (1.000–1.000)
**MLST CC**	0.010 (0.000–0.028)	0.997 (0.995–1.000)	0.157 (0.076–0.238)	0.997 (0.995–1.000)	0.389 (0.253–0.524)	

^a^Numbers inside the parenthesis represent 95% confidence intervals

^b^MT, MLVA type

^c^ST, Sequence type

The high genetic diversity observed by MLVA among serotypes 14, 9N and 9V isolates in the present study complies with the fact that such serotypes are frequently found associated with different clinical conditions, ranging from asymptomatic nasopharyngeal colonization to severe invasive infections [4–9], indicating that different strains have evolved rapidly and independently due to different selective pressures; thus, generating an elevated number of unrelated clones expressing the same capsular type. Conversely, previous studies that have applied MLVA for serotype 1 isolates have shown that they represent homogeneous populations, usually represented by a few number of highly related MTs, regardless of the VNTR panel used or the origin of the isolates, which is in accordance with the observation that such serotypes are rarely found in nasopharyngeal carriage, being specialized in causing invasive diseases, especially meningitis outbreaks [19, 37, 38].

The discriminatory power of each one of the 18 loci was also evaluated (Table 3). Spneu36 presented the highest index followed by Spneu37 and Spneu25. Similarly, previous studies [23, 24] have described high SIDs (above 80%) for loci Spneu37 and Spneu25. However, Spneu36 was only previously evaluated by Pichon et al. [20], who reported that this locus was among the seven most discriminatory loci for invasive serotype 5 isolates. As stated previously [39], higher diversity indexes are usually observed for those VNTR loci presenting a higher interval or average in the number of repeats. Moreover, the location of each locus along the bacterial genome can also have an impact on its genetic diversity, since it defines the mechanisms and levels of selective pressure that are active on such sites, changing the mutation model and rate [36]. Although VNTR loci are presumably neutral markers, they can be near other loci that are under selective pressure, such as the *psp*C gene in the case of Spneu36 and *psp*A gene in the case of Spneu25 [39, 40].

**Table 3:** Indexes of diversity observed for each one of the 18 VNTR loci evaluated in the present study for 87 *Streptococcus pneumoniae* isolates belonging to serotypes 14, 9N and 9V.

VNTR locus^a^	Number of alleles^b^	SID^c^ (95% CI)
**Spneu 15**	9	0.737 (0.678–0.795)
**Spneu 17**	7	0.700 (0.650–0.749)
Spneu 19	5	0.427 (0.313–0.542)
**Spneu 25**	5	0.740 (0.702–0.779)
Spneu 26	5	0.446 (0.330–0.561)
Spneu 27	4	0.637 (0.585–0.689)
Spneu 31	4	0.650 (0.610–0.689)
Spneu 32	4	0.369 (0.250–0.488)
**Spneu 33**	6	0.700 (0.644–0.756)
Spneu 34	4	0.462 (0.376–0.549)
Spneu 35	4	0.611 (0.552–0.670)
**Spneu 36**	8	0.828 (0.801–0.856)
**Spneu 37**	9	0.760 (0.697–0.824)
Spneu 38	4	0.542 (0.447–0.637)
**Spneu 39**	8	0.725 (0.676–0.773)
Spneu 40	6	0.713 (0.663–0.763)
Spneu 41	4	0. 539 (0.476–0.601)
Spneu 42	5	0.729 (0.678–0.781)

^a^VNTR selected in the present study to propose a short MLVA scheme are shown in bold

^b^Number of different alleles observed among 87 *Streptococcus pneumoniae* isolates of serotype 14 and serogroup 9

^c^SID, Simpson’s Index of Diversity; 95% CI, Confidence Interval.

**Fig 3:**
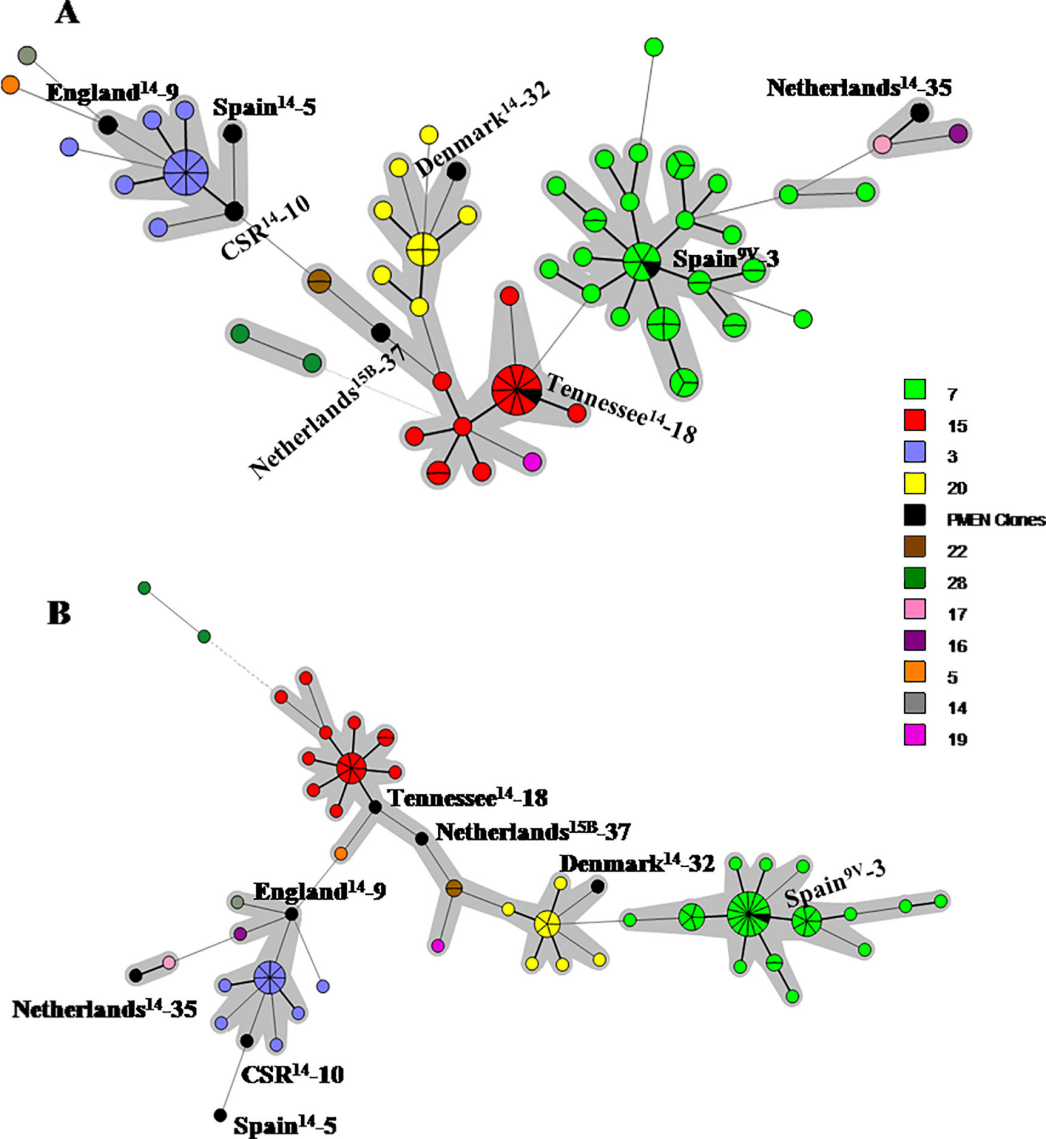
**Minimum Spanning Tree showing the genetic relationship observed by MLVA among 87 *Streptococcus pneumoniae* strains of serotypes 14, 9N and 9V and the eight PMEN clones included in the present study using the VNTR panels proposed by (A) Pichon et al. [20] and (B) van Cuyck et al. [22].** Legend indicates the MLVA clonal complexes determined with the 18 VNTR loci panel analysis. Each circle represents one MLVA type, and the size of the circle indicates the number of isolates belonging to the same type. PMEN clones are described. Clonal complexes are highlighted with a grey shadow. Lines linking the circles denote the genetic distance as follows: heavy short lines connect single locus variants (SLVs), thin longer lines connect double-locus variants (DLVs) and dotted lines indicate difference by 3 or more loci.

**Fig 4:**
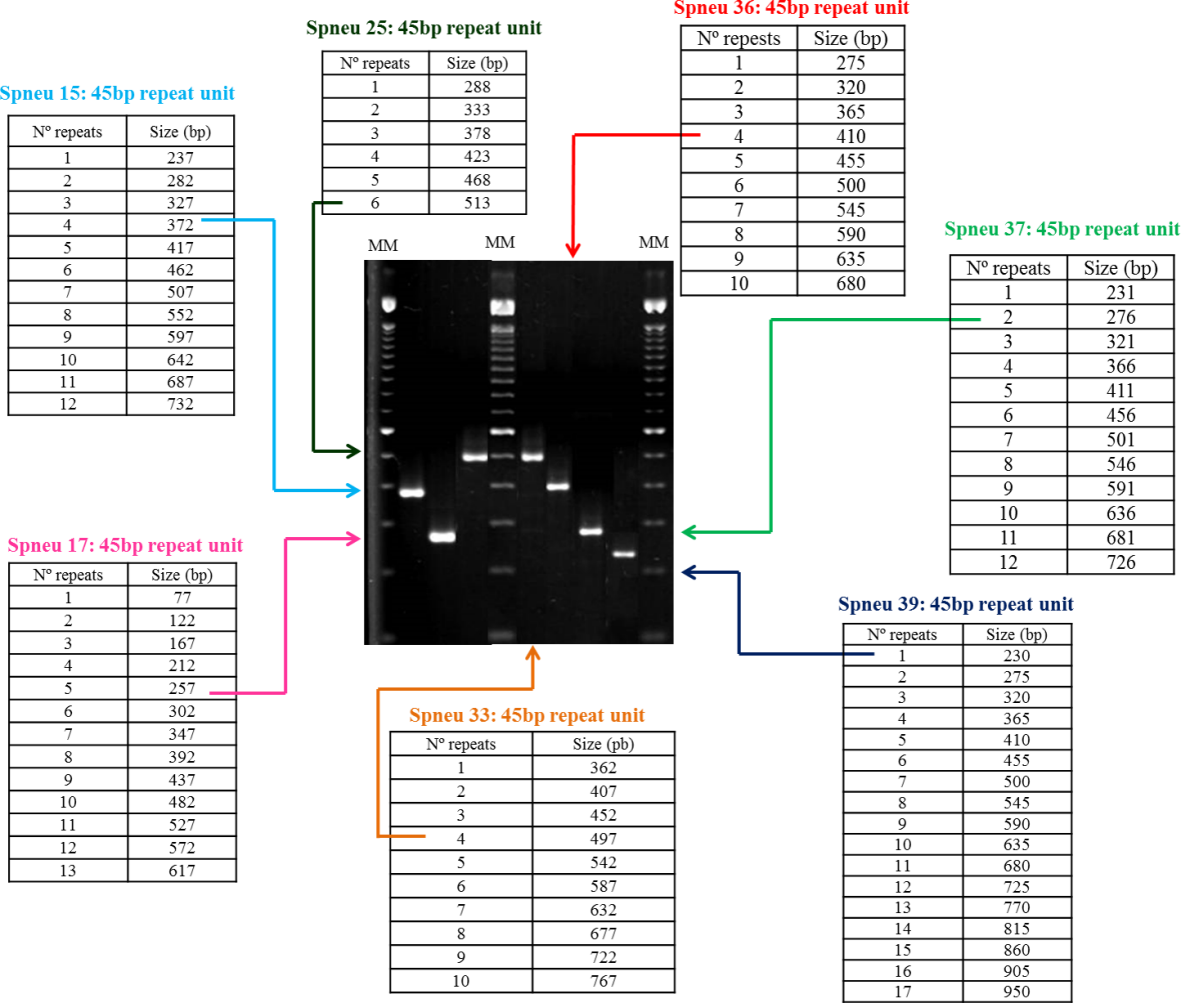
Schematic representation of the 7 VNTR loci selected in the present study, including the range of repeat numbers and estimated size of amplicons, to compose a novel reduced panel for MLVA typing of *Streptococcus pneumoniae*.

Different sets of markers were already proposed for a universal MLVA scheme [20–23]. For comparative purposes, the 87 pneumococcal strains belonging to serotypes 14, 9N and 9V included in the present study, in addition to the eight PMEN clones, were also analyzed using two of those previously recommended MLVA reduced panels (Table 4). These two previously described panels were selected for comparison since they included the same number of loci (total of 7) and were based exclusively on the 18 loci originally identified by Koeck et al. [19], such as the present study. Both of them, however, have clustered isolates belonging to different MLST CCs in the same MLVA CC (Fig 3). Therefore, 7 VNTR loci were selected to constitute a novel condensed proposal (Table 4 and Fig 4). This selection took into account, besides the SID of each locus, the convenience associated with the estimation of the number of repeats by using solely agarose gel electrophoresis. In this regard, locus Spneu42, which contains a repeat unit of 14bp, was excluded as option despite the high SID observed (Table 3).

**Table 4:** *In silico* comparative evaluation between the reduced panel of VNTR loci suggested in the present study and reduced panels previously proposed for MLVA typing of *Streptococcus pneumoniae* isolates, for 87 *Streptococcus pneumoniae* strains of serotypes 14, 9N and 9V and 8 PMEN clones.

Proposed panel	Number of loci included	Selected VNTR loci among those previously identified by Koeck et al. [19] Spneu																		Number of genotypes^a^	SID^b^	AW^c^
		15	17	19	25	26	27	31	32	33	34	35	36	37	38	39	40	41	42			
Pichon et al. [20]	7		X	X	X						X		X	X		X				50	0.972 (0.958–0.986)	0.707 (0.642–0.773)
van Cuyck et al. [22]	7		X	X	X		X			X				X		X				45	0.954 (0.933–0.975)	0.674 (0.605–0.743)
This study	7	X	X		X					X			X	X		X				54	0.979 (0.966–0.991)	1.000 (1.000–1.000)

^a^Among 87 *Streptococcus pneumoniae* strains of serotypes 14, 9N and 9V and 8 PMEN clones included in the present study

^b^SID, Simpson’s Index of Diversity

^c^AW, Adjusted Wallace Coefficient showing the congruence level with the sequence type (ST) determined by MLST.

**Fig 5:**
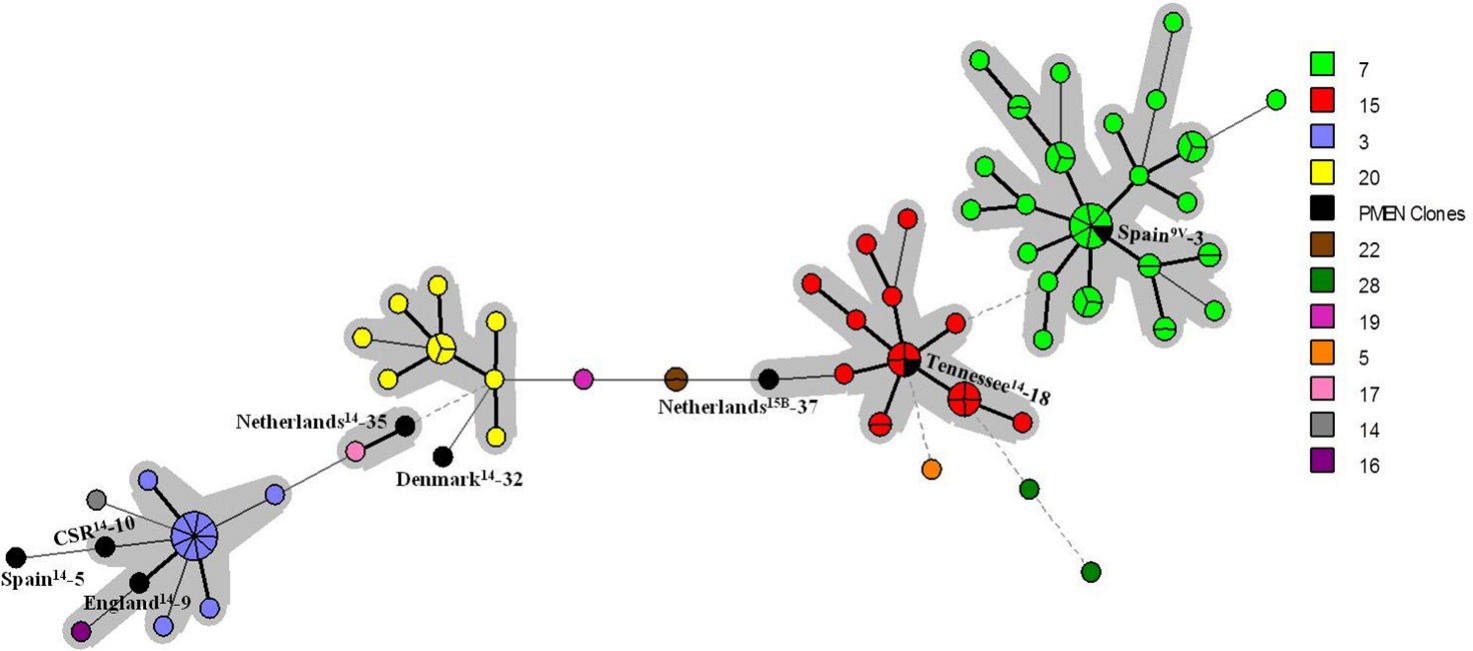
Minimum Spanning Tree showing the genetic relationship observed by MLVA among 87 *Streptococcus pneumoniae* strains of serotypes 14, 9N and 9V and the eight PMEN clones included in the present study using the proposed reduced panel of 7 VNTR loci. Legend indicates the MLVA clonal complexes determined with the 18 VNTR loci panel analysis. Each circle represents one MLVA type, and the size of the circle indicates the number of isolates belonging to the same type. PMEN clones are described. Clonal complexes are highlighted with a grey shadow. Lines linking the circles denote the genetic distance as follows: heavy short lines connect single locus variants (SLVs), thin longer lines connect double-locus variants (DLVs) and dotted lines indicate difference by 3 or more loci.

The analysis of the same isolates performed by using this reduced MLVA panel revealed 54 MTs distributed among four CCs and eight singletons (Fig 5). The CCs detected with the complete 18 loci MLVA panel were maintained apart with the newly proposed scheme, as were also conserved the associations between PMEN clones and respective CCs. The compact scheme showed a SID of 97.9% (Table 4), indicating a high discriminatory power and preserving the advantage of MLVA over the other methods evaluated (PFGE and MLST) regarding the diversity index.

Comparing the reduced MLVA schemes proposed to date [20–23] with the one of the present study, loci Spneu25 and Spneu37 are the only ones shared by all, probably because they usually present high SIDs regardless of the collection of pneumococcal isolates analyzed, indicating that such loci would be suitable for composing a more stable and universal MLVA protocol to genotype pneumococcal isolates. The set of VNTR markers, whose composition depends on the bacterial population studied, could be used either to investigate local outbreaks or to track the worldwide spread of clones and particularly the emergence of variants. In general, highly diverse VNTR loci would allow increased discrimination in short-term studies, whereas VNTR loci with low diversity would allow identification of long-term changes. It has been suggested that, for epidemiological studies, the best panel of molecular markers should comprise several highly discriminatory loci plus a poorly discriminatory one [22]. The approach of using only moderately discriminatory loci, as observed for some previously proposed panels [20, 22], is not ideal since they are neither discriminatory enough to accurately separate the CCs nor slightly diverse in order to correctly cluster strains in the same CC.

**Fig 6:**
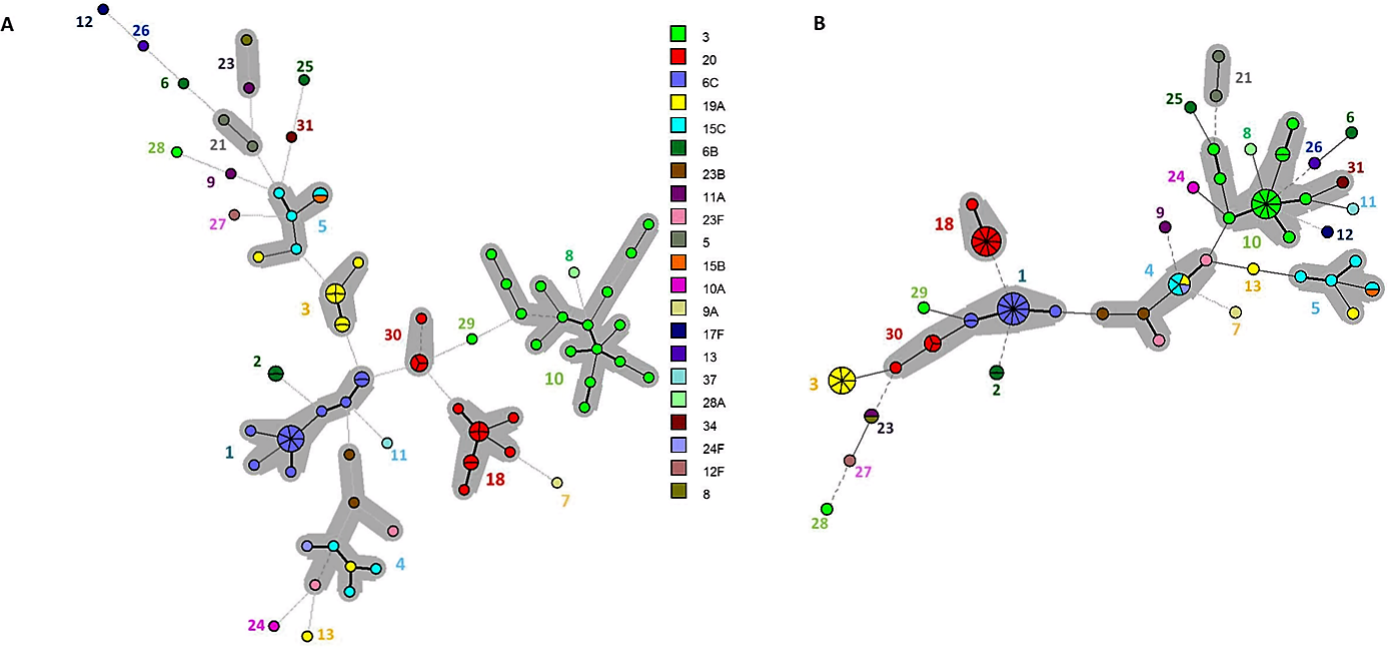
**A. Minimum Spanning Tree showing the genetic relationship observed by MLVA using the complete panel of 18 loci among 87 pneumococcal strains belonging to different serotypes. B. Minimum Spanning Tree showing the genetic relationship observed by MLVA using the reduced proposed panel of 7 loci among 87 pneumococcal strains belonging to different serotypes**. Different MLVA CCs are highlighted. Serotypes are differentiated by colors and displayed in subtitles.

**Fig 7:**
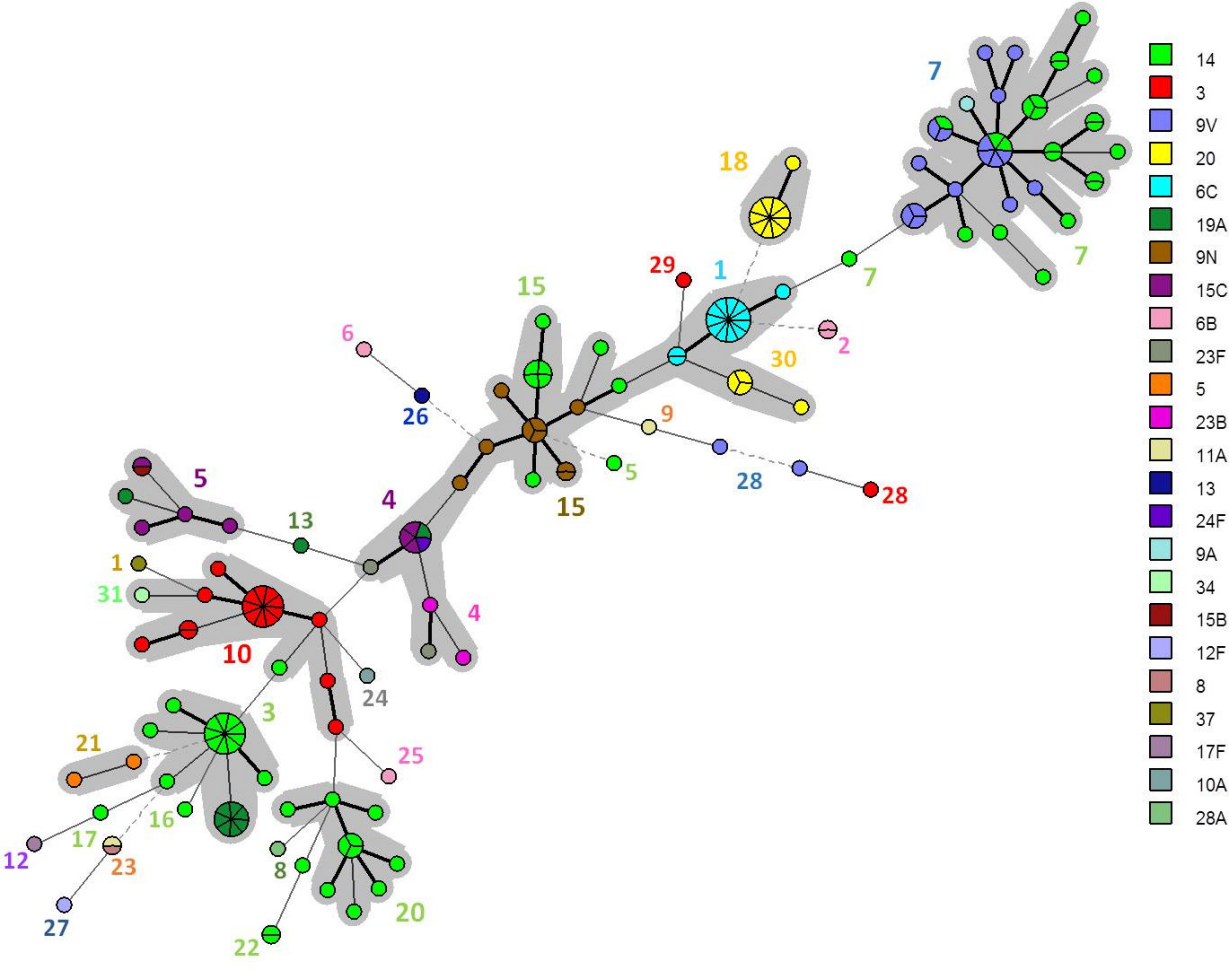
Minimum Spanning Tree showing the genetic relationship observed by MLVA using the reduced proposed panel of 7 loci among all the 174 pneumococcal isolates investigated. Different MLVA CCs are highlighted. Serotypes are differentiated by colors and displayed in subtitles.

In order to pre-evaluate the new scheme of 7 VNTR loci proposed here, 87 pneumococcal isolates belonging to distinct serotypes were also examined in the present study. Using all the 18 VNTR loci, it was possible to detect nine CCs and fifteen singletons, with a diversity index of 99%. CCs comprised isolates belonging to the same or related serotypes (Fig 6A). Using the condensed 7 VNTR loci MLVA scheme, CCs were maintained (Fig 6B) and the diversity index was 95.6%, indicating that the newly proposed panel may also be a reliable and discriminatory tool to genotype pneumococcal isolates of different serotypes, besides those belonging to serotypes 14, 9N and 9V. When all the 174 pneumococcal isolates were analyzed together, diversity index was higher than 98% (Fig 7). Nonetheless, in order to validate the short MLVA scheme proposed on the basis of the results generated in the present work, a broader evaluation including pneumococcal isolates recovered from other countries should be performed.

In conclusion, all the pneumococcal isolates included in the present study, which represented asymptomatic carriage and IPD cases occurring during a long period of time in Brazil, were typeable by MLVA, being the newly proposed panel of 7 VNTR loci highlighted for the high discriminatory power, besides significantly reducing the amount of reagents, time and laboratory workload. The results also showed high congruence of MLVA with PFGE and MLST, indicating that it constitutes a faster and cost-effective alternative for predicting MLST clonal complexes within this important bacterial species.

The findings indicated that the 7 loci MLVA scheme constitutes a more practical and reliable alternative for typing pneumococcal isolates and a potential candidate for establishing a simplified universally applicable MLVA method for *S*. *pneumoniae*, that will facilitate the comparison of results obtained in different laboratories and may encounter potential for large-scale applications, especially in resource-limited areas, since it does not require any sophisticated equipment or software besides conventional PCR and agarose gel electrophoresis.

## Supporting Information

S1 Table Characteristics and MLVA profiles of all *Streptococcus pneumoniae* isolates analyzed in the present study. (XLSX)
